# Significance of definitive concurrent chemoradiotherapy for vulvar cancer: a Japanese Gynecologic Oncology Group nationwide survey study

**DOI:** 10.1007/s11604-024-01557-9

**Published:** 2024-04-03

**Authors:** Noriyuki Okonogi, Keisuke Tsuchida, Ken Ando, Tatsuya Ohno, Hiroyuki Fujiwara, Kosuke Yoshihara, Takuya Aoki, Hirokuni Takano, Munetaka Takekuma, Aikou Okamoto, Shin Nishio

**Affiliations:** 1 Department of Radiation Oncology, Juntendo University Graduate School of Medicine, 2-1-1 Hongo, Bunkyo-Ku, Tokyo, 113-8421 Japan; 2 Department of Radiation Oncology, Kanagawa Cancer Center, 2-3-2 Nakao, Asahi-Ku, Yokohama, 241-8515 Japan; 3 Department of Radiation Oncology, Gunma University Graduate School of Medicine, 3-39-22 Showa-Machi, Maebashi, Gunma 371-8511 Japan; 4 Department of Obstetrics and Gynecology, Jichi Medical University, 3311-1 Yakushiji, Shimotsuke-Shi, Tochigi, 329-0498 Japan; 5 Department of Obstetrics and Gynecology, Niigata University Graduate School of Medical and Dental Sciences, 1-757 Asahimachidori, Chuo-Ku, Niigata City, Niigata 951-8510 Japan; 6 Department of Obstetrics and Gynecology, Kobe City Medical Center General Hospital, 2-1-1 Minatojima Minamimachi, Chuo-Ku, Kobe City, Hyogo 650-0047 Japan; 7 Department of Gynecologic Oncology, The Jikei University School of Medicine, 3-25-8, Nishi-Shimbashi, Minato-Ku, Tokyo, 105-8461 Japan; 8 Department of Gynecologic Oncology, Shizuoka Cancer Center, 1007 Shimonagakubo, Nagaizumi-Cho, Sunto-Gun, Shizuoka Prefecture 411-8777 Japan; 9 Department of Obstetrics and Gynecology, Kurume University School of Medicine, 67 Asahi-Machi, Kurume, Fukuoka 830-0011 Japan

**Keywords:** Vulvar cancer, Radiotherapy, Concurrent chemoradiotherapy, Nationwide survey

## Abstract

**Objective:**

This study aimed to show the results of radical radiation therapy (RT) and concurrent chemoradiotherapy (CCRT) for vulvar cancer (VC) based on data from a Japanese nationwide survey.

**Materials and methods:**

We collected data from 108 institutions on cases of VC diagnosed between January 2001 and December 2010. Patients with histologically proven squamous cell carcinoma and adenocarcinoma with curative intent were selected, and 172 patients with VC were included in this study. The collected data were analyzed for overall survival (OS) using the Kaplan–Meier method. Univariate and multivariate analyses were performed to examine the prognostic factors for patients with VC.

**Results:**

The median follow-up period was 16.8 (range; 3.2–154.8) months. Fifty-five patients received CCRT**,** and 117 patients received RT alone. The 2-year OS rates (95% confidence interval [CI]) for stages I, II, III, and IV were 77.9% (55.8–100.0), 71.9% (53.8–89.9), 55.4% (42.5–68.3), and 41.5% (27.3–55.7) respectively. Univariate analyses showed that the FIGO stage (*p* = 0.001), tumor diameter (*p* = 0.005), and lymph node (LN) status (*p* = 0.001) were associated with OS. The concurrent use of chemotherapy resulted in a significantly longer OS in Stage III (*p* = 0.013). Multivariate analysis showed that the hazard ratios (95% CI) for tumor diameter, positivity for LN metastasis, and RT alone (no concurrent chemotherapy) were 1.502 (1.116–2.021), 1.801 (1.287–2.521), and 1.936 (1.187–3.159), respectively.

**Conclusions:**

Our analysis revealed that CCRT should be recommended, especially for Stage III VC patients. Further studies are warranted to determine who benefits from CCRT, considering primary tumor size and LN status.

The study was registered at the University Hospital Medical Information Network (protocol number: UMIN000017080) on April 8th, 2015.

## Introduction

Vulvar cancer (VC) is a rare gynecological disease. There is a 30-fold variation in incidence rates worldwide, with the highest reported in South African data (age-standardized incidence rates; ASR, 7.2 per 100,000 inhabitants). However, in Western Asia and the Middle East, this disease is uncommon (ASR, 0.2 per 100,000 inhabitants) [1]. Its incidence is 0.4 per 100,000 inhabitants in Japan and is expected to increase with the aging of the population [2].

Surgical therapy is the primary option for patients with resectable VC [3]. Radiation therapy (RT) serves as an adjuvant treatment following initial surgery, as part of the primary therapy for locally advanced disease, or for palliation purposes in cases of recurrent/metastatic disease [3]. Several studies have reported on the significance of RT as an adjuvant treatment for VC. According to the GOG37 trial, adjuvant RT improved the survival of patients with clinically suspicious or fixed ulcerated groin nodes and two or more positive groin nodes [4]. A retrospective study showed that adjuvant RT significantly improves the survival of patients with positive surgical margins [5].

In contrast to adjuvant RT, the available evidence regarding the efficacy of definitive RT for patients with VC remains limited. Indeed, several studies demonstrated the efficacy and tolerability of concurrent chemoradiotherapy (CCRT) for VC [6–8]. However, the findings presented in these reports were derived from data collected from a few dozen patients or from phase II trials. Therefore, stage-specific outcomes and efficacy of combination chemotherapy in patients with VC have not been fully evaluated.

Here, we analyzed the results of radical RT/CCRT for VC on a larger scale, based on data from a nationwide Japanese survey. We also analyzed the significance of concomitant chemotherapy for each stage of the disease, as well as the prognostic factors for patients with VC.

## Materials and methods

### Study design

This was a sub-analysis of a multicenter, retrospective, observational study. The protocol for this study was approved by the ethics committee of each participating institution (108 Japanese Gynecologic Oncology Group [JGOG] affiliated institutions) [9]. The study was registered with the University Hospital Medical Information Network (UMIN) (protocol number: UMIN000017080). Consecutive VC cases diagnosed between January 2001 and December 2010 were eligible. The inclusion criteria for the original study were as follows: histologically proven and treated VC, including patients whose initial therapy was of palliative intent, and cases of primary vulvar cancer with all histologic types, with the exception of malignant melanoma. The details are summarized in our previous study [9]. The data were collected from 108 JGOG-affiliated institutions between August 2014 and March 2016, and included stage, histology, treatment intensity (curative or palliative), treatment modality (surgery, RT, chemotherapy), RT dose, and survival periods.

### Criteria of this study

**Fig. 1:**
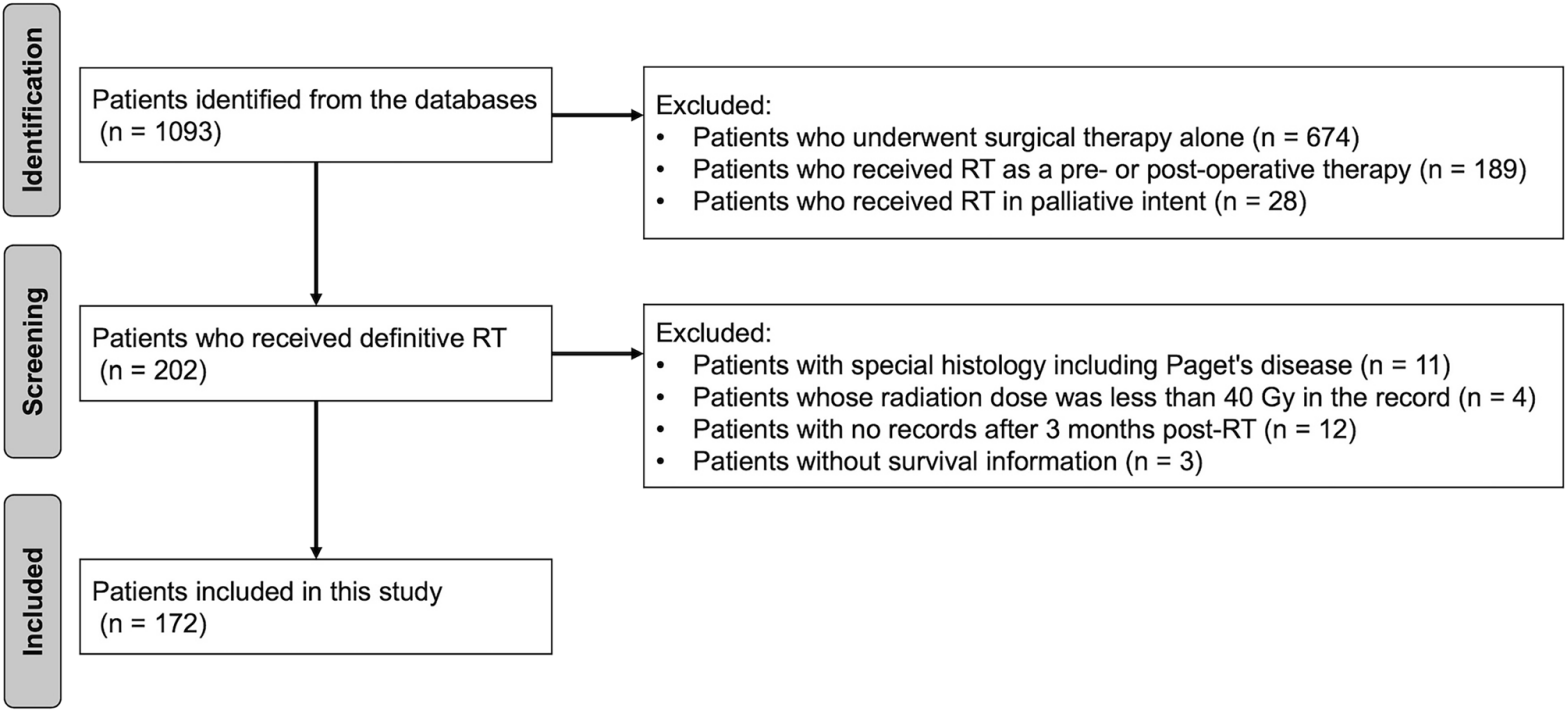
Patient selection criteria. It shows a flowchart of patient selection for the present study. *RT* radiation therapy

This study aimed to clarify the clinical significance of radical RT/CCRT in patients with VC using the patient cohort of the original study. Therefore, we first excluded from the original data VC patients who had undergone surgery or were reported to be in palliative care. Next, in order to increase the reliability of the data in this study, patients with the following items were excluded: (i) patients with special histology, including Paget's disease, (ii) patients whose radiation dose was less than 40 Gy in the record, (iii) patients with no records after three months post-RT, and (iv) patients without survival information. The “40 Gy” setting in this study was intended to exclude obvious cases of palliative irradiation. Figure 1 shows a flowchart of the patient selection process. Of the patients identified in the database, 172 with VC were included in this study. In this study, patients who received at least one course of concurrent chemotherapy were defined to be in the CCRT group.

### Statistical analysis

The collected data were analyzed for overall survival (OS) using the Kaplan–Meier method. OS was defined as the time from the initiation of RT/CCRT to death from any cause. Univariate analyses were performed using the log-rank test. Regarding cutoffs for univariate analyses, median values were used for age and tumor diameter. For the cutoff value of the total dose, we adopted 60 Gy, which is the minimum value recommended by the NCCN guidelines to control gross primary disease [3]. Multivariate analysis was performed using a Cox proportional hazards regression model. The analysis was conducted on the variables that exhibited a p-value of less than 0.1 in the univariate analysis. Confounding factors were assessed and stratified before the multivariate analysis. The magnitude of the effect was expressed as a hazard ratio (HR) and 95% confidence interval (CI). The level of statistical significance was set at *p* < 0.05. All statistical tests were two-sided. Statistical analyses were performed using the IBM SPSS Statistics version 27 (IBM, Armonk, NY, USA).

## Results

**Fig. 2:**
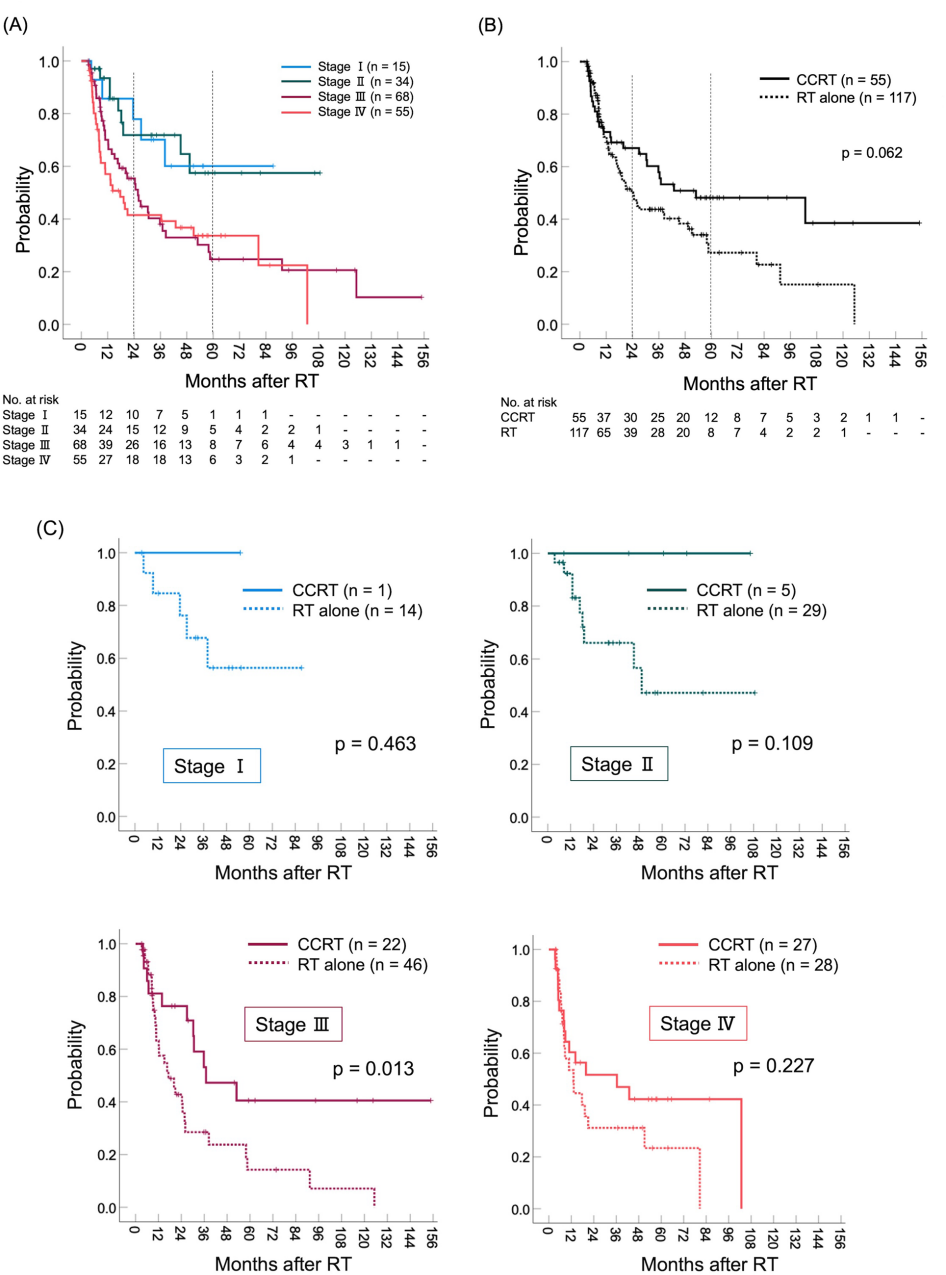
Overall survival curves stratified by FIGO staging. **A** Shows the Kaplan–Meier curves of OS stratified by FIGO staging. **B** Shows the Kaplan–Meier curves of OS stratified by the use of concurrent chemotherapy (RT versus CCRT). **C** Shows the OS curves for RT and CCRT at each stage. Solid lines indicate CCRT and dashed lines indicate RT in **B** and **C**. *OS* overall survival, *FIGO* International Federation of Gynecology and Obstetrics, *RT* radiation therapy, *CCRT* concurrent chemoradiotherapy

Table 1 shows the patient and tumor characteristics that met the eligibility criteria for this study. The median follow-up periods were 16.8 months (range; 3.2–154.8 months) in all patients and 18.4 months (range; 3.2–108.6 months) in the surviving patients. The classification of histologic types whose patients analyzed in this study included only squamous cell carcinoma (165 patients) and adenocarcinoma (7 patients) and did not include patients with other histologic types. Fifty-five patients underwent CCRT, 54 of whom received platinum-based combination chemotherapy. The remaining patient received 5-fluorouracil (5-FU) combination chemotherapy. Of 55 patients who underwent CCRT, one patient received adjuvant chemotherapy. Figure 2A shows the Kaplan–Meier curves of OS stratified by the International Federation of Gynecology and Obstetrics (FIGO) staging. The 2-year OS rates (95% CI) in Stage I, II, III, and IV were 77.9% (55.8–100.0), 71.9% (53.8–89.9), 55.4% (42.5–68.3), and 41.5% (27.3–55.7) respectively. The 5-year OS rates (95% CI) in Stage I, II, III, and IV were 60.1% (32.3–88.0), 57.5% (34.5–80.4), 24.7% (11.9–37.6), and 33.7% (19.6–47.7) respectively. Figure 2B shows the OS curves stratified by the use of concurrent chemotherapy (RT versus CCRT). The 2-year and 5-year OS rates (95% CI) in CCRT group were 67.1% (57.6–76.3) and 48.2% (33.7–62.7), respectively. Whereas, the 2-year and 5-year OS rates (95% CI) in RT alone group were 50.2% (39.8–60.5) and 27.2% (15.0–39.4), respectively. Figure 2C shows the OS curves for RT and CCRT at each stage. CCRT resulted in a higher OS rate than RT alone at all stages. A statistically significant difference was observed in the superiority of CCRT in patients with Stage III (p = 0.013).

**Table 1:** Patient and tumor characteristics who met the eligibility (n = 172)

Characteristic	n (range or %)
Age at diagnosis (years), median (range)	76 (42–95)
FIGO stage (2008)	
I	15 (8.7)
II	34 (19.8)
III	68 (39.5)
IV	55 (32.0)
Histology	
Squamous cell carcinoma	165 (95.9)
Adenocarcinoma	7 (4.1)
Tumor diameter (mm), median (range)	48 (8–150)
Tumor diameter (mm)	
< 48	65 (37.8)
≥ 48	66 (38.4)
N/R	41 (23.8)
LN status	
Negative for LN metastasis	35 (20.3)
Positive for LN metastasis	53 (30.8)
N/R	84 (48.8)
Number of positive LNs	
0	35 (20.3)
1	19 (11.0)
2	16 (9.3)
≥ 3	18 (10.5)
N/R	84 (48.8)

*FIGO* the International Federation of Gynecology and Obstetrics, *N/R* not reported, *LN* lymph node, *Gy* Gray

The prognostic factors assessed using univariate analysis are summarized in Table 2. FIGO stage (*p* = 0.001), tumor diameter (*p* = 0.005), and lymph node (LN) status (*p* = 0.001) were associated with OS. The concurrent use of chemotherapy showed a trend of better prognosis, although the difference was not statistically significant (p = 0.062). No RT-related factors (irradiation volume, irradiation method, or total dose) were associated with OS.

**Table 2:** Assessment of prognostic factors with univariate analysis

Factor	Number of patients	OS		
		2-year (%)	5-year (%)	*p* value
Age (years)				
< 76	83	55.6	38.3	0.972
≥ 76	89	57.5	31.8	
FIGO stage (2008)				
I + II	49	74.2	58.0	0.001
III + IV	123	49.3	28.6	
Histology				
Squamous cell carcinoma	165	56.9	36.6	0.455
Adenocarcinoma	7	41.7	20.8	
Tumor diameter (mm)				
< 48	65	70.1	39.9	0.005
≥ 48	66	40.5	25.3	
LN status				
Negative for LN metastasis	35	83.7	66.4	0.001
Positive for LN metastasis	53	42.9	24.5	
Irradiation volume				
Local disease	30	55.0	32.8	0.755
Local and regional	126	56.0	37.0	
Irradiation method				
X-ray alone	129	55.8	35.8	0.889
X-ray and electron boost	31	58.9	31.6	
Total dose (Gy)				
< 60	57	51.4	45.4	0.648
≥ 60	98	58.7	33.2	
Concurrent use of chemotherapy				
No	117	50.2	27.2	0.062
Yes	55	67.1	48.2	

*OS* overall survival, *FIGO* the International Federation of Gynecology and Obstetrics, *LN* lymph node

Evaluation of confounding factors revealed a confounding relationship between stage and tumor diameter and between Stage and LN status. Thus, a multivariate analysis of OS was conducted using tumor diameter, LN status, and concurrent chemotherapy. As shown in Table 3, all these items were associated with OS. The HRs (95% CI) for tumor diameter ≥ 48 mm, positive for LN metastasis, and RT alone (no concurrent chemotherapy) were 1.502 (1.116–2.021), 1.801 (1.287–2.521), and 1.936 (1.187–3.159), respectively.

**Table 3:** Assessment of prognostic factors with multivariate analysis

Factor	Number of deaths	OS	
		*p* value	HR (95% CI)
Tumor diameter ≥ 48 mm	39	0.007	1.502 (1.116–2.021)
Positive for LN metastasis	34	0.001	1.801 (1.287–2.521)
RT alone (no concurrent chemotherapy)	62	0.008	1.936 (1.187–3.159)

*OS* overall survival, *HR* hazard risk, *CI* confidence intervals, *LN* lymph node, *RT* radiation therapy

## Discussion

This study represents an investigation that validates the importance of RT/CCRT for VC and demonstrates its effectiveness in a cohort of over 100 cases. To our knowledge, few studies on RT/CCRT in patients with VC with a large number of patients have been reported to date. In our study, the 5-year OS rates for stages I, II, III, and IV were 60.1%, 57.5%, 24.7%, and 33.7%, respectively. Considering that the median age of the population was 76 years, and the combined Stage III and IV population was over 70%, RT/CCRT for VC would be an effective treatment option. In accordance with the Japanese Nationwide Study, the 5-year OS rates of patients with stage I, II, III, and IV VC who underwent surgical treatment were 85.6%, 75.1%, 48.8%, and 40.0%, respectively [9]. These results suggest that, if tumors are medically resectable, surgical procedures should be recommended first.

Our study also indicated the significance of administering concurrent chemotherapy for the management of patients with VC. This study provides evidence for the superiority of CCRT over RT, particularly in patients in Stage III. Therefore, CCRT should be considered unless the organ functions are compromised. As shown in Fig. 2C, a statistically significant difference might have been observed if there had been a larger number of patients other than Stage III. However, the present study did not allow a detailed analysis by primary tumor size or LN status at each stage. Further analysis of the groups that benefit from CCRT in terms of survival is warranted. When combining chemotherapy and RT, several chemotherapeutic regimens have been reported for patients with VC [10–13]. Since the late 1990s, the combination of 5-FU and Mitomycin C with RT has been examined in patients with VC [10–12]. In recent years, regimens similar to those for cervical cancer, including platinum-based chemotherapy, have been examined [13]. A retrospective study based on a database survey also reported the efficacy of cisplatin-based CCRT for VC patients [14]. Currently, the NCCN recommends cisplatin or 5-FU/cisplatin as the CCRT regimen for VC [3]. To draw conclusions about the treatment-related quality of life and adverse events, prospective studies on these chemotherapy regimens are required.

Larger tumor diameter, positive LN metastasis, and the absence of concurrent chemotherapy were negatively associated with OS in this study. This supports the findings reported by Rao et al. [14]. A previous retrospective review demonstrated that larger tumor size and positive LN metastasis were independent poor prognostic factors in surgically treated VC patients [15]. Another study showed that groin LN metastasis was an independent prognostic factor for patient survival in T1 and T2 VC patients who underwent surgery [16]. Although we were unable to analyze the location of LN metastasis in our study, tumor size and LN status certainly seem to be important even in radical RT for VC patients. However, no association was observed between RT-related factors (irradiation dose, irradiation method, or total dose) and OS. Lalliscia et al. reported that a total dose of > 54 Gy was associated with a lower risk of disease progression and death in an analysis of adjuvant RT [17]. Stecklein et al. reported a 3-year OS rate of 51% in patients with macroscopic diseases who received high-dose RT (median, 66 Gy; range 60–70 Gy) [18]. However, Rishi et al. reported that a dose > 66 Gy was a predictor of severe toxicity, even with high-precision RT such as intensity-modulated RT [19]. Thus, although the optimal dose has not yet been clearly defined, radiation dose is an important factor. In RT for perineal tumors, such as VC, severe acute and late adverse events significantly compromise the quality of life. Arrangements of the RT dose that consider tumor spread and risk of toxicity are warranted in clinical settings.

The present study had several limitations. The first limitation is its retrospective nature. Thus, we could not analyze the impact of operability on OS or provide a reason why surgical treatment was not chosen in patients who received radical RT. Other clinical endpoints, such as local control, recurrence-free survival, and toxicities, could not be evaluated due to substantial missing data. The short observation period is also a limitation of this study. Given the variability in post-treatment follow-up durations across different facilities, a shorter median observation period may be inherent in a retrospective observational study. Another limitation of this study is lack of detailed information on RT technique such as use of brachytherapy. A multicenter, prospective observational study is required to overcome these limitations.

In conclusion, we reported the significance of definitive CCRT for VC. Our analysis revealed that CCRT should be recommended, especially for stage III VC patients. Further studies are warranted to determine who benefits from CCRT, considering primary tumor size and LN status.
